# Soluble Epoxide Hydrolase Inhibitor and 14,15-Epoxyeicosatrienoic Acid-Facilitated Long-Term Potentiation through cAMP and CaMKII in the Hippocampus

**DOI:** 10.1155/2017/3467805

**Published:** 2017-08-24

**Authors:** Han-Fang Wu, Yi-Ju Chen, Su-Zhen Wu, Chi-Wei Lee, I-Tuan Chen, Yi-Chao Lee, Chi-Chen Huang, Chung-Hsi Hsing, Chih-Wei Tang, Hui-Ching Lin

**Affiliations:** ^1^Department and Institute of Physiology, School of Medicine, National Yang-Ming University, Taipei, Taiwan; ^2^Department of Anesthesiology, Chi-Mei Medical Center, Tainan, Taiwan; ^3^Graduate Institute of Neural Regenerative Medicine, College of Medical Science and Technology, Taipei Medical University, Taipei, Taiwan; ^4^Department of Neurology, Far Eastern Memorial Hospital, New Taipei City, Taiwan; ^5^Institute of Brain Science, National Yang-Ming University, Taipei, Taiwan; ^6^Brain Research Center, National Yang-Ming University, Taipei, Taiwan

## Abstract

Epoxyeicosatrienoic acids (EETs) are derived from arachidonic acid and metabolized by soluble epoxide hydrolase (sEH). The role of EETs in synaptic function in the central nervous system is still largely unknown. We found that pharmacological inhibition of sEH to stabilize endogenous EETs and exogenous 14,15-EET significantly increased the field excitatory postsynaptic potential (fEPSP) response in the CA1 area of the hippocampus, while additionally enhancing high-frequency stimulation- (HFS-) induced long-term potentiation (LTP) and forskolin- (FSK-) induced LTP. sEH inhibitor (sEHI) N-[1-(oxopropyl)-4-piperidinyl]-N'-[4-(trifluoromethoxy) phenyl)-urea (TPPU) and exogenous 14,15-EET increased HFS-LTP, which could be blocked by an N-methyl-D-aspartate (NMDA) receptor subunit NR2B antagonist. TPPU- or 14,15-EET-facilitated FSK-mediated LTP can be potentiated by an A1 adenosine receptor antagonist and a phosphodiesterase inhibitor, but is prevented by a cAMP-dependent protein kinase (PKA) inhibitor. sEHI and 14,15-EET upregulated the activation of extracellular signal-regulated kinases (ERKs) and Ca2+/calmodulin- (CaM-) dependent protein kinase II (CaMKII). Phosphorylation of synaptic receptors NR2B and *α*-amino-3-hydroxy-5-methyl-4-isoxazolepropionic acid (AMPA) receptor subunit GluR1 was increased by TPPU and 14,15-EET administration. These results indicated that EETs increased NMDAR- and FSK-mediated synaptic potentiation via the AC-cAMP-PKA signaling cascade and upregulated the ERKs and CaMKII, resulting in increased phosphorylation of NR2B and GluR1 in the hippocampus.

## 1. Introduction

Epoxyeicosatrienoic acids (EETs) are epoxide derivatives formed from arachidonic acid (ARA). Cytochrome P450 (CYP) epoxygenases of CYP2C and CYP2J subfamilies actively metabolize ARA to various EETs, including 5,6-, 8,9-, 11,12-, and 14,15-EETs. EETs have been shown to be potent vasodilators and to exert anti-inflammatory, antithrombotic, and antioxidant effects; they are also important regulators of the cardiovascular system. EETs are metabolized via soluble epoxide hydrolase (sEH) to form dihydroxyeicosatrienoic acids (DHETs) [1]. Stabilization of EETs through inactivation of sEH, pharmacological inhibition of sEH, and sEH deletion is an attractive therapeutic target in many conditions and diseases, including pain, hypertension, inflammation, and ischemia [1]. Animal studies have demonstrated that EETs play roles in synaptic function, depression, and Parkinsonism [2–4]. Additionally, 14,15-EET is a regioisomer that is hydrolyzed with the highest Vmax from sEH. sEH^−/−^ mice also have a higher level of 14,15-EET in the brain [5]. A growing body of evidence suggests that 14,15-EET is implicated in the regulation of physiological functions of endothelial cells, such as angiogenesis [6, 7]. In cortical neurons, 14,15-EET promotes axon outgrowth. Furthermore, increasing levels of 14,15-EET enhance the viability of astrocytes and prevent brain damage during ischemic injury [8]. So far, no study has examined the effects of exogenous and endogenous 14,15-EETs in terms of regulating synaptic activity and synapse function.

The hippocampus is a major region of the brain that mediates memory, such as spatial memory, and cognitive behavior. Long-term potentiation (LTP) is well known as the cellular basis of learning and memory [9–11]. Impaired LTP in the CA1 region of hippocampus slices commonly occurs in aged transgenic mouse models of Alzheimer's disease [12, 13]. Exogenous EETs 14,15-EET or 11,12-EET have been shown to prevent amyloid *β*-induced mitochondria dysfunction in cultured hippocampal astrocytes [14]. These previous studies raise the possibility that exogenous EETs might play a role in the modulation of LTP in the CA1 region of the hippocampus. Classically, LTP can be triggered by electrical high-frequency stimulation (HFS) and chemically induced by the adenylyl cyclase activator forskolin [15, 16]. LTP occurs in the induction phase to trigger rapid potentiation dependent on phosphorylation of preexisting proteins and in the maintenance phase to sustain the potentiation response dependent on new protein synthesis [17–19]. We have previously demonstrated that the sEH inhibitor AUDA enhances synaptic transmission [4]. In this study, we examined whether exogenous 14,15-EET administration and pharmacologic inhibition of sEH to stabilize endogenous EETs influenced HFS-induced LTP and forskolin-induced LTP. We hypothesized that elevated levels of EETs will promote electrically induced LTP and chemically induced LTP (cLTP). Furthermore, we determined the possible mechanisms underlying LTP enhancement by EETs in the induction phase and maintenance phase.

## 2. Materials and Methods

### 2.1. Animals

All procedures were approved by the Institutional Animal Care and Use Committee of the College of Medicine, National Yang-Ming University (Taipei, Taiwan). Eight-week-old C57BL/6 mice were used in this study. They were housed to a cage in a temperature-controlled (24°C) animal colony under a 12 : 12 light/dark cycle, with lights on at 7:00 AM. Pelleted chow and water were available ad libitum. All experimental procedures took place during the light cycle.

### 2.2. Brain Slice Preparation and Electrophysiological Recordings of the Hippocampus

400 *μ*m coronal slices containing the hippocampal region were prepared from mouse brains. After hippocampal region preparation, slices were placed in a chamber in artificial cerebral spinal fluid (ACSF) solution (saturated with 95% O_2_ and 5% CO_2_) and maintained at room temperature for at least 1 h before obtaining electrophysiological recordings. The ACSF was of the following composition (in mM): NaCl 117, KCl 4.7, CaCl_2_ 2.5, MgCl_2_ 1.2, NaHCO_3_ 25, NaH_2_PO_4_ 1.25, and glucose 11 [20]. Then, slices were transferred to a recording chamber, in which they were continually perfused with oxygenated ACSF (30–32°C). To record the field excitatory postsynaptic potential (fEPSP) in the hippocampus, a concentric bipolar stimulating electrode (FHC, Bowdoinham, ME, USA) was evoked at the Schaffer collateral/commissural afferent at 0.033 Hz and a capillary glass recording electrode filled with 3 M NaCl solution was placed in the CA1 stratum radiatum [21]. HFS-induced LTP was elicited by a 3× HFS protocol (100 Hz for 1 s at 20 s intervals). The chemical LTP induction solution consisted of the above ACSF and 25 *μ*M forskolin (Tocris, St. Louis, MO, USA). After 10 minutes of induction of chemical LTP and subsequent washing with ACSF for approximately 5 minutes, the fEPSP was monitored for an hour. Chemically induced LTP (cLTP) was induced by forskolin in the hippocampal brain slices.

### 2.3. Drugs

N-[1-(1-Oxopropyl)-4-piperidinyl]-N'-[4-(trifluoromethoxy)phenyl)-urea (TPPU), (±)14(15)-epoxy-5Z,8Z,11Z-eicosatrienoic acid (14,15-EET), 20-hydroxy-5Z,8Z,11Z,14Z-eicosatetraenoic acid (20-HETE), 8-cyclopentyl-1,3-dipropylxanthine (DPCPX), 2-chloroadenosine (2-CADO), and 4-(3-butoxy-4-methoxyphenyl)methyl-2-imidazolidone (Ro 20-1724) were obtained from Cayman. Forskolin and Rp-Cyclic 3′,5′-hydrogen phosphorothioate adenosine (Rp-cAMPs), (*αR*,*βS*)-*α*-(4-hydroxyphenyl)-*β*-methyl-4-(phenylmethyl)-1-piperidinepropanol maleate (Ro 25-6981) and (9R,10S,12S)-2,3,9,10,11,12-hexahydro-10-hydroxy-9-methyl-1-oxo-9,12-epoxy-1H-diindolo[1,2,3-fg:3′,2′,1′-kl]pyrrolo[3,4-i][1,6]benzodiazocine-10-carboxyl-ic acid, and hexyl ester (KT 5720) were obtained from Tocris. Stock solutions of ACSF and dimethyl sulfoxide (DMSO) were prepared. The concentration of DMSO did not exceed 0.1% and had no effect on basal synaptic transmission. In the present study, the control was 0.1% DMSO in ACSF.

### 2.4. Western Blotting Assay

Brain tissues were dissected and lysed in a lysis buffer containing 1% Triton X-100, 0.1% SDS, 50 mM Tris-HCl, pH 7.5, 0.3 M sucrose, 5 mM EDTA, 2 mM sodium pyrophosphate, 1 mM sodium orthovanadate, and 1 mM enylmethylsulfonyl fluoride, supplemented with a complete protease inhibitor cocktail. Following sonication, lysates were centrifuged at 12,000 rpm for 30 min to obtain supernatants. The protein concentrations of the supernatants were measured using a Bradford assay, and equal amounts of protein were separated by SDS-PAGE electrophoresis, transferred to Immobilon-P membranes (Millipore), and incubated in 5% nonfat dry milk for 60 min. Western blot analysis was performed using GluR1 (1 : 1000; Abcam), CaKMII (1 : 1000; Abcam), ERK (1 : 2000; Cell Signaling), NR2B (1 : 2000; Abcam), GAPDH (1 : 10,000; Abcam), *p*-NR2B (1 : 1000; Abcam), *p*-GluR1 (1 : 1000; Abcam), *p*-CaKMII (1 : 1000; Cell Signaling), and *p*-ERK (1 : 2000; Cell Signaling) antibodies, reacted overnight at 4°C, and then incubated with HRP-conjugated secondary antibodies for 1 h at room temperature. Immunoreactivity was detected using ECL Plus detection reagent (PerkinElmer, Boston, MA). Films were exposed for different durations to ensure optimum density but were not saturated, and densitometry was then performed. Protein levels were first normalized to the internal control level for each sample and then measured as fold changes with respect to the controls.

### 2.5. Statistical Analysis

All values are expressed as the mean ± SEM. Electrophysiologic responses and the protein levels of the control, TPPU-, 14,15-EET-, and 20-HETE-exposed groups and LTP were analyzed by one-way ANOVA followed by Bonferroni's post hoc test. Differences between treatment groups were considered significant if *p* < 0.05.

## 3. Results

### 3.1. Effects of sEHI, 14,15-EETs, and 20-HETE on Hippocampal Neurons

**Figure 1:**
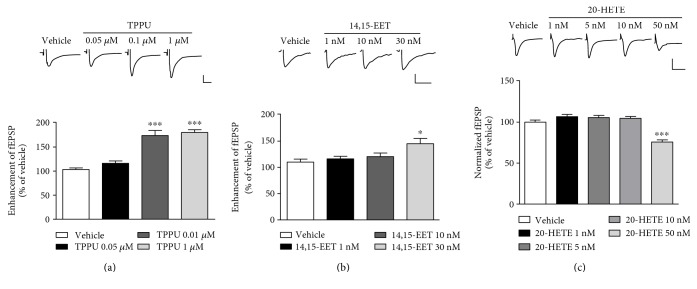
Acute TPPU and 14,15-EET applications increased excitatory synaptic transmission at the Schaffer collateral-CA1 hippocampal synapses. (a) Effects of TPPU (0.05, 0.1, and 1 *μ*M) administration on fEPSP response. (b) Effects of 14,15-EET (1, 10, and 30 nM) administration on fEPSP response. (c) Effects of 20-HETE (1, 5, 10, and 50 nM) administration on fEPSP response. Data represent means ± SEM in each experiment (*n* = 5 from 5 mice). ^∗^*p* < 0.05, ^∗∗∗^*p* < 0.001 compared with vehicle group; scale, 40 ms and 0.5 mV.

To examine the effect of TPPU on basal excitatory synaptic transmission, we measured the synaptic response by recording the fEPSP in area CA1 of the hippocampus. A dose-related effect of TPPU was observed when the hippocampus slices were superfused with different doses (0.05 *μ*M, 0.1 *μ*M, and 1 *μ*M). As shown in Figure 1(a), one-way ANOVA demonstrated that TPPU at concentrations of 0.1 *μ*M (173.9 ± 10.5%, *n* = 6 from 5 mice, *p* < 0.001 versus vehicle) and 1 *μ*M (180.4 ± 5.7%, *n* = 6 from 5 mice, *p* < 0.001 versus vehicle) increased the synaptic response in terms of the fEPSP (F_(3,20)_ = 39.7, *p* < 0.001; Figure 1(a)).

We further determined that the synaptic response was affected by 14,15-EET treatment. Hippocampus slices were superfused with various doses of 14,15-EET (1 nM, 10 nM, and 30 nM), which resulted in a significantly increased fEPSP slope at 30 nM 14,15-EET (145.1 ± 10.9%, *n* = 6 from 5 mice, *p* < 0.05 versus vehicle). One-way ANOVA showed a significant main effect (F_(3,20)_ = 4.6, *p* < 0.05) (Figure 1(b)).

Evidence has shown that ARA is metabolized through CYP enzymes to EETs and DHETs [22, 23]. To examine whether basal excitatory synaptic transmission is affected by 20-HETE treatment, hippocampus slices were superfused with various doses of 20-HETE (1 nM, 5 nM, 10 nM, and 50 nM). There were no differences in the fEPSP slope between the vehicle, 1 nM, 5 nM, and 10 nM 20-HETE groups (F_(3,20)_ = 1.8, *p* > 0.05 versus vehicle). An unexpected result was that 20-HETE at a dose of 50 nM resulted in inhibition of the fEPSP slope (F_(4,25)_ = 32.5, *p* < 0.001 versus vehicle) (Figure 1(c)). These results demonstrated that sEH inhibitor (sEHI) TPPU increased the endogenous EET level in the hippocampus, and TPPU and exogenous 14,15-EET, but not 20-HETE, enhanced excitatory synaptic transmission.

### 3.2. TPPU and 14,15-EET Facilitated HFS-Induced LTP

**Figure 2:**
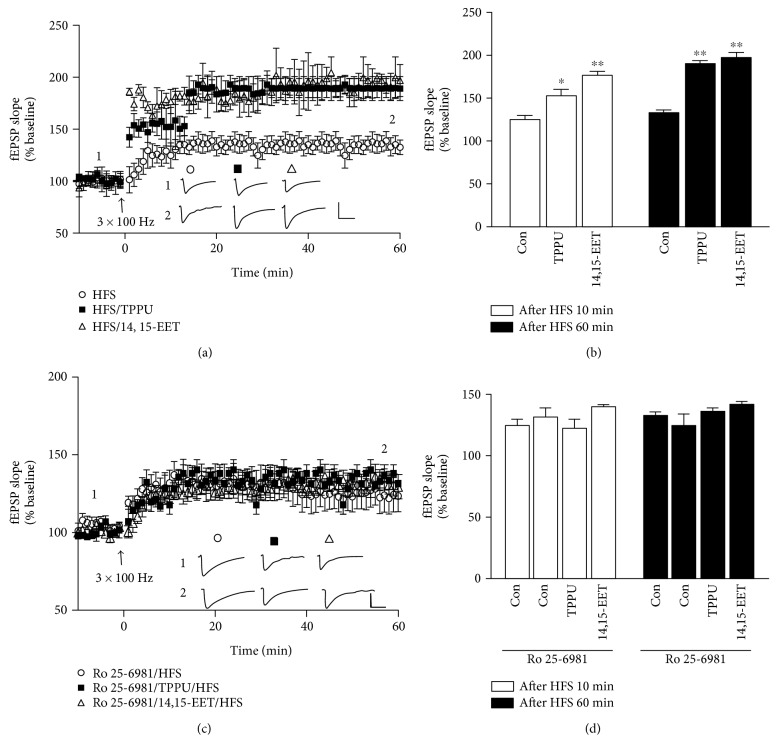
TPPU- and 14,15-EET-facilitated HFS-induced LTP are blocked by NR2B antagonist in the hippocampus. (a) Effects of TPPU (0.1 *μ*M) and 14,15-EET (30 nM) on the enhancement of HFS-induced LTP. (b) Bar chart comparing the effects of TPPU and 14,15-EET after potentiation for 10 min and 60 min. (c) Administration of Ro 25-6981 (1 *μ*M) blocked TPPU- and 14,15-EET-enhanced HFS-induced LTP. (d) Bar chart showing that Ro 25-6981 blocked TPPU- and 14,15-EET-enhanced HFS-induced LTP after potentiation for 10 min and 60 min. Data represent means ± SEM in each experiment (*n* = 6). ^∗^*p* < 0.05, ^∗∗^*p* < 0.01; scale, 40 ms and 0.5 mV.

To evaluate the impact of TPPU and 14,15-EET on the induction of LTP at the hippocampal synapses, we applied HFS-induced (3 times for 1 sec at 100 Hz stimuli separated by intervals of 20 sec) LTP of Schaffer collateral-CA1 synapses. As shown in Figure 2(a), incubation of hippocampal slices with TPPU enhanced HFS-induced LTP (F_(2,15)_ = 19.44, *p* < 0.001). In addition, the degree of HFS-induced LTP was also enhanced in the presence of 14,15-EET (30 nM) during the LTP induction and maintenance phases (Figure 2(a)). We further compared the effects in terms of induction and maintenance on HFS-induced LTP of TPPU and 14,15-EET treatments after 10 min (control: 124.7 ± 7.4% of baseline, *n* = 6 from 5 mice; TPPU: 176.6 ± 9.7% of baseline, *n* = 6 from 5 mice, *p* < 0.05; and 14,15-EET: 152.4 ± 5.7% of baseline, *n* = 6 from 5 mice, *p* < 0.01) and 60 min (control: 132.6 ± 8.4% of baseline, *n* = 6 from 5 mice; TPPU: 176.9 ± 7.9% of baseline, *n* = 6 from 5 mice, *p* < 0.01; and 14,15-EET: 172.4 ± 9.7% of baseline, *n* = 6 from 5 mice, *p* < 0.01) (Figure 2(b)). We attempted to examine whether the role of NR2B-containing NMDA receptors contributed to TPPU- and 14,15-EET-facilitated LTP. We first confirmed that HFS-induced LTP was affected by an NR2B-NMDAR antagonist in the hippocampal CA1 region. In agreement with previous findings [24, 25], bath incubation of a selective NMDA receptor NR2B antagonist, Ro 25-6981 (1 *μ*M), did not block HFS-induced LTP in the hippocampal CA1 region (*t*_(10)_ = 0.73, *n* = 6 from 5 mice, *p* = 0.73) (Figures 2(c) and 2(d)). There were no differences in the normalized fEPSP slope after HFS for 10 mins between Ro 25-6981/HFS, Ro 25-6981/TPPU/HFS, and Ro 25-6981/14,15-EET/HFS (Ro 25-6981/HFS: 131.4 ± 8.9% of baseline, *n* = 6 from 5 mice; Ro 25-6981/TPPU/HFS: 122.3 ± 4.7% of baseline, *n* = 6 from 5 mice; and Ro 25-6981/14,15-EET/HFS: 139.9 ± 7.7% of baseline, *n* = 6 from 5 mice). Similar results were obtained in the LTP maintenance phase, in that there were no differences in the normalized fEPSP slope after HFS for 60 mins between Ro 25-6981/HFS, Ro 25-6981/TPPU/HFS, and Ro 25-6981/14,15-EET/HFS (Ro 25-6981/HFS: 124.3 ± 9.6% of baseline, *n* = 6 from 5 mice; Ro 25-6981/TPPU/HFS: 135.8 ± 2.6% of baseline, *n* = 6 from 5 mice; and Ro 25-6981/14,15-EET/HFS: 141.9 ± 3.8% of baseline, *n* = 6 from 5 mice). These results demonstrated that TPPU (F_(2,15)_ = 1.96, *p* > 0.5) and 14,15-EET (F_(2,15)_ = 2.15, *p* > 0.5) failed to facilitate LTP in the presence of an NMDA receptor NR2B antagonist (Figures 2(c) and 2(d)). Thus, NR2B NMDARs contribute to TPPU- and 14,15-EET-facilitated LTP in hippocampal slices.

### 3.3. TPPU and 14,15-EET Enhanced Forskolin- (FSK-) Induced LTP

**Figure 3:**
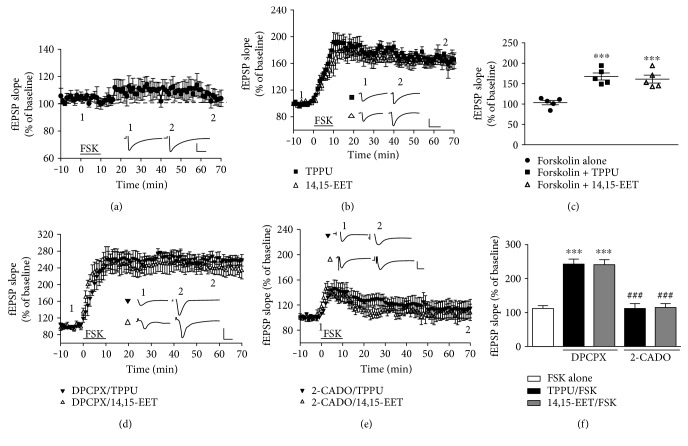
TPPU and 14,15-EET facilitated FSK-induced LTP. (a) Effect of FSK (25 *μ*M) on the fEPSP response in hippocampal neurons. (b) Effects of TPPU and 14,15-EET on FSK-induced LTP. (c) Comparison of the effects shown in (a) and (b). (d) Effects of TPPU and 14,15-EET on FSK-induced LTP in the presence of DPCPX (500 nM). (e) Effects of TPPU and 14,15-EET on FSK-induced LTP in the presence of 2-CADO (1 *μ*M). (f) Bar chart comparing the effects of TPPU and 14,15-EET on FSK-induced LTP in the presence or absence of DPCPX and 2-CADO. Data represent means ± SEM in each experiment (*n* = 6). ^∗∗∗^*p* < 0.001 versus FSK alone. ^###^*p* < 0.001 versus DPCPX group; scale, 40 ms and 0.5 mV.

We examined the effects of TPPU and 14,15-EET on cLTP in hippocampal slices by application of forskolin and found that a low concentration of forskolin (25 *μ*M) had no significant effect on LTP induction, which was similar to the results of previous studies (107.5 ± 6.1% of baseline, *n* = 6; Figures 3(a) and 3(c)) [26]. Interestingly, a low concentration of forskolin could induce LTP in the presence of TPPU and 14,15-EET (forskolin + TPPU: 167.3 ± 9.5% of baseline, *n* = 5 from 3 mice; *p* < 0.001; forskolin + 14,15-EET: 161.7 ± 10.8% of baseline, *n* = 5 from 3 mice; *p* < 0.001; Figures 3(b) and 3(c)). These results suggested that TPPU and 14,15-EET promote FSK-mediated synaptic potentiation (F_(2,12)_ = 19.3, *p* < 0.001 versus FSK alone) (Figure 3(c)). We determined whether adenosine receptor involvement in TPPU and 14,15-EET promoted FSK-mediated synaptic potentiation. Slices were preincubated with selective adenosine receptor antagonist 8-cyclopentyl-1,3-dipropylxanthine (DPCPX). Figures 3(d) and 3(f) show that TPPU (*n* = 5 from 4 mice) and 14,15-EET (*n* = 5 from 4 mice) facilitation of FSK-mediated synaptic potentiation were promoted by DPCPX (500 nM) (F_(2,10)_ = 33.7, *p* < 0.001). However, adenosine receptor agonist 2-chloroadenosine (2-CADO) at 1 *μ*M reversed TPPU (*n* = 5 from 4 mice) and 14,15-EET (*n* = 5 from 4 mice) promotion of FSK-mediated synaptic potentiation (F_(3,16)_ = 28.9, *p* < 0.001) (Figures 3(e) and 3(f)).

### 3.4. TPPU- and 14,15-EET-Enhanced Forskolin- (FSK-) Induced LTP Is Mediated by cAMP-PKA Signaling

**Figure 4:**
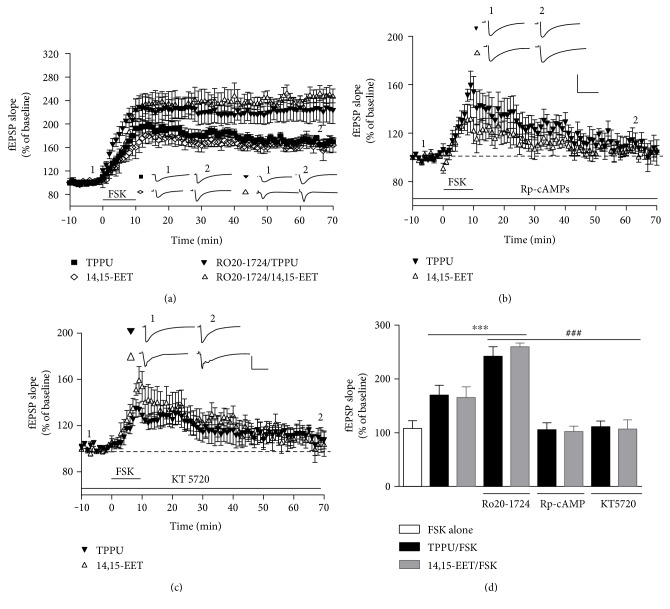
The cAMP-PKA pathway is involved in TPPU- and 14,15-EET-facilitated forskolin-induced synaptic potentiation. (a) Effects of TPPU and 14,15-EET on forskolin-induced LTP in the presence of Ro 20-1724 (10 *μ*M). (b) Effects of TPPU and 14,15-EET on forskolin-induced LTP in the presence of Rp-cAMPs (25 *μ*M). (c) Effects of TPPU and 14,15-EET on forskolin-induced LTP in the presence of KT 5720 (1 *μ*M). (d) Bar chart comparing the effects of TPPU and 14,15-EET on forskolin-induced LTP in the presence or absence of Ro 20-1724, Rp-cAMPs, and KT 5720. Data represent means ± SEM in each experiment (*n* = 6). ^∗∗∗^*p* < 0.001 versus TPPU/FSK, 14,15-EET/FSK; ^###^*p* < 0.001 versus Ro 20-1724 group; scale, 40 ms and 0.5 mV.

It has been demonstrated that cAMP can be hydrolyzed by phosphodiesterase (PDE); therefore, we evaluated whether elevation of cAMP by PDE inhibitor contributes to TPPU and 14,15-EET promotion of FSK-mediated synaptic potentiation. Slices were pretreated with cAMP-specific phosphodiesterase inhibitor Ro 20-1724 (10 *μ*M) (*n* = 6 from 5 mice in each group). As shown in Figures 4(a) and 4(d), TPPU and 14,15-EET promotion of FSK-mediated LTP was dramatically enhanced by Ro 20-1724 (F_(3,20)_ = 32.7, *p* < 0.001). To further investigate whether forskolin/TPPU- and forskolin/14,15-EET-induced LTP potentiation were mediated through activation of cAMP-dependent protein kinase (PKA), hippocampal slices were preincubated with specific PKA regulatory site antagonist Rp-cAMPS (25 *μ*M) (*n* = 6 from 5 mice in each group) or PKA inhibitor KT5720 (1 *μ*M). The results presented in Figures 4(b) and 4(d) showed that the effects of TPPU and 14,15-EET in terms of enhancing forskolin-induced LTP were blocked by Rp-cAMPS (F_(3,20)_ = 3.9, *p* < 0.05). Similar results were obtained under treatment with PKA inhibitor (*n* = 6 from 5 mice in each group), in that TPPU and 14,15-EET failed to promote FSK-mediated potentiation in the presence of KT5720 (F_(3,20)_ = 28.6, *p* < 0.001) (Figures 4(c) and 4(d)).

### 3.5. TPPU and 14,15-EET Increased Phosphorylation of NMDA Receptor NR2B Subunit and AMPA Receptor GluR1

**Figure 5:**
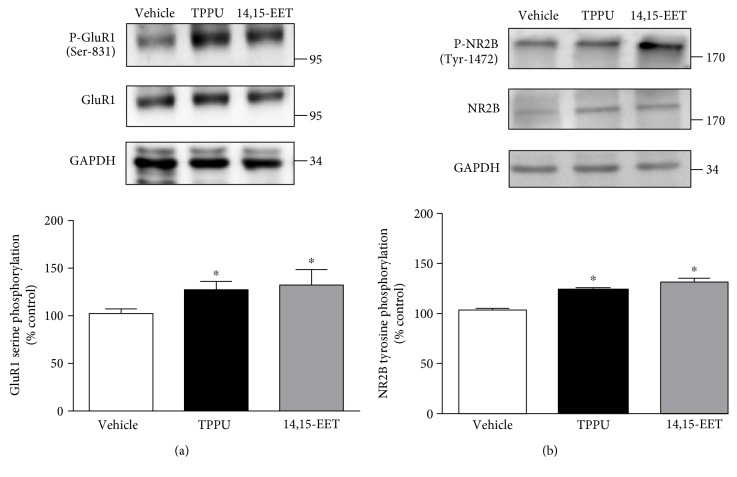
Phosphorylation of glutamate receptors in the hippocampus was increased by TPPU and 14,15-EET. (a) Hippocampal slices were incubated with TPPU and 14,15-EET for 20–25 min and then washed. Homogenate from the hippocampus was prepared and blotted with antibodies to serine 831 (Ser-831) phosphorylation in the GluR1 subunit of the AMPA receptor and total GluR1 subunit of the AMPA receptor and (b) Tyr-1472 phosphorylation in the NR2B subunit of the NMDA receptor and total NR2B subunit of the NMDA receptor. Data represent means ± SEM in each experiment. ^∗^*p* < 0.05.

To assess whether AMPA receptor phosphorylation and NMDA receptor phosphorylation are involved in the regulation of LTP by treatment with TPPU and 14,15-EET, Western blot analysis was performed. Treatment with TPPU and 14,15-EET significantly increased the phosphorylation of NR2B (F_(2,12)_ = 26.9, *p* < 0.001) and GluR1 (F_(2,12)_ = 9.8, *p* < 0.01) in hippocampal slices. GluR1 phosphorylation (126.8 ± 9.1% of vehicle, *n* = 5 from 5 mice) and NR2B phosphorylation (123.2 ± 5.1% ± 7.1%, *n* = 5 from 5 mice) both increased in the presence of TPPU. A similar result was achieved with application of 14,15-EET, in terms of increased GluR1 phosphorylation and NR2B phosphorylation (132.9 ± 7.9% of vehicle, *n* = 5 from 5 mice; 131.2 ± 5.4% of vehicle, *n* = 5 from 5 mice; Figure 5).

### 3.6. TPPU and 14,15-EET Increased Activation of ERK and CaMKII

**Figure 6:**
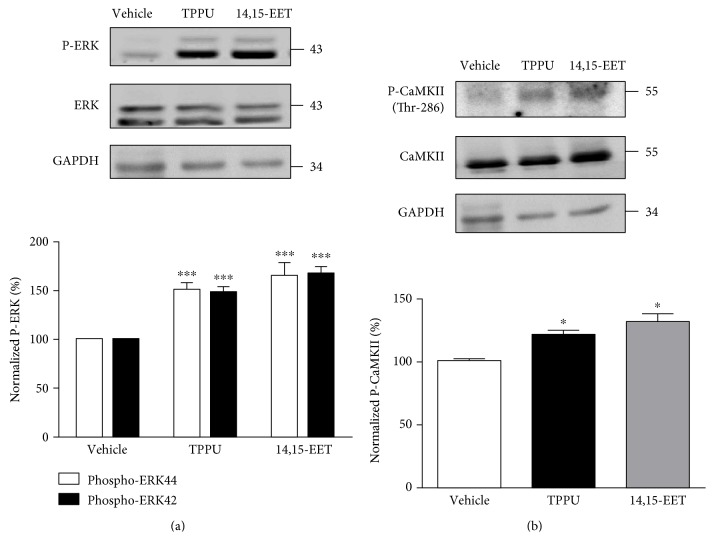
Phosphorylation of ERK and CaMKII in the hippocampus was increased by TPPU and 14,15-EET. (a) Hippocampal slices were incubated with 14,15-EET and TPPU for 20–25 min and then washed. Homogenate from the hippocampus was prepared and blotted with antibodies to ERK phosphorylation and (b) Thr-286 phosphorylation in CaMKII. Data represent means ± SEM in each experiment. ^∗^*p* < 0.05, ^∗∗∗^*p* < 0.001.

**Figure 7:**
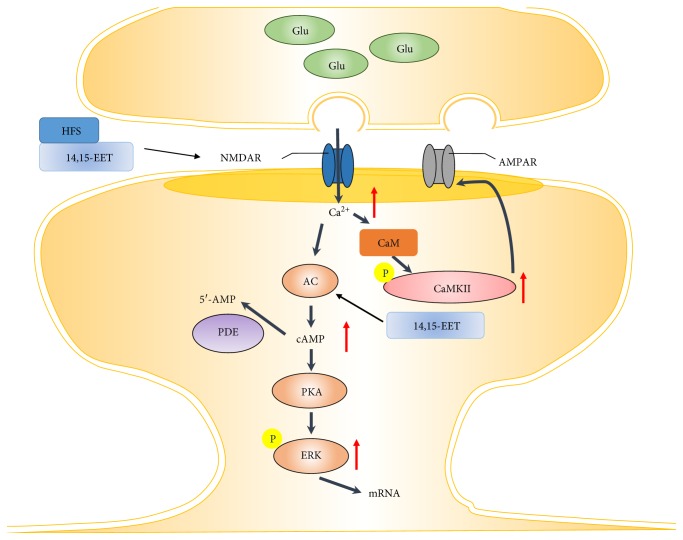
Schematic diagram of the mechanism of sEHI and 14,15-EET promotion of the LTP process via activation of CaMKII, PKA, and ERK in the hippocampus.

We further investigated the mechanism involved in the regulation of LTP by TPPU and 14,15-EET treatments (Figure 6()). We examined the phosphorylation of ERK and CaMKII in hippocampal slices. According to the results, in the presence of TPPU, phosphorylation of ERK42 (148.5 ± 5.9% of vehicle, *n* = 6 from 6 mice) and ERK44 (150.7 ± 5.8% of vehicle, *n* = 6 from 6 mice) and CaMKII phosphorylation were increased as compared with the vehicle control (122.2 ± 5.1% of vehicle, *n* = 6 from 6 mice). 14,15-EET treatment also led to significant increases in the phosphorylation of ERK42 (143.5 ± 5.9% of vehicle, *n* = 6 from 6 mice), ERK44 (141.7 ± 5.8% of vehicle, *n* = 6 from 6 mice), and CaMKII (132.2 ± 6.4% of vehicle, *n* = 5 from 5 mice). Treatment with TPPU and 14,15-EET resulted in significant increases in the levels of phospho-ERK42 (F_(2,15)_ = 18.3, *p* < 0.05), phospho-ERK44(F_(2,15)_ = 27.9, *p* < 0.05), and phospho-CaMKII (F_(2,12)_ = 7.4, *p* < 0.05). These results demonstrated that 14,15-EET promotes the LTP process via activation of CaMKII, PKA, and ERK in hippocampus (Figure 7()).

## 4. Discussion

The findings of this study suggested that inhibition of sEH by TPPU and 14,15-EET administration could modulate the synaptic response in the hippocampus area. We demonstrated that both TPPU and 14,15-EET increased both electrically induced LTP and cLTP in the induction phase and maintenance phase. An A1 adenosine antagonist and an elevated cAMP level by a PDE inhibitor enhanced the TPPU- and 14,15-EET-mediated glutamatergic synaptic plasticity. NR2B and PKA antagonists blocked sEHI- and 14,15-EET-facilitated LTP. TPPU and 14,15-EET upregulated *p*-ERK and *p*-CaMKII. Meanwhile, phosphorylation of NR2B and GluR1 was increased by sEHI and 14,15-EET treatments. Our findings provided evidence that HFS-induced LTP and cLTP at the hippocampal synapses could be regulated by 14,15-EET.

Arachidonic acid is converted to EETs by CYP epoxygenases, that is, CYP 2C and CYP 2J, whereas ARA is converted to 20-HETE by CYP *ω*-hydroxylase, that is, CYP 4A and CYP 4F [22, 23]. Our result showed that the level of 14,15-EET was increased following inhibition of sEH in hippocampal slices. An elevated 20-HETE level in the urinary system has been reported in mice lacking the sEH gene [27]. Thus, TPPU, 14,15-EET, and 20-HETE may regulate glutamate-mediated synaptic transmission in the hippocampus. Our results demonstrated that TPPU and 14,15-EET potentiated glutamate-mediated synaptic transmission, whereas 20-HETE at less than 10 nM did not change the basal response of the fEPSP slope, while 20-HETE at 50 nM decreased the fEPSP slope in the hippocampal CA1 region. Interestingly, opposite effects of EETs and 20-HETE on large-conductance calcium-activated K^+^ channels in renal microvascular smooth muscle cells have been reported [28]. Low concentrations of 20-HETE have a slight inward rectification effect, whereas high concentrations have an outward rectification effect on the transient receptor potentiation cation channel subfamily C member 6 (TRPC6 channel) in HEK 293 cells [29]. Evidence has also shown that sEH gene deletion and sEH inhibitor have opposite effects on the cardiac system [30]. The reasons for the different effects of EETs and 20-HETE at the hippocampal synapses require further investigation.

In addition, key CYP is a major synthesized enzyme of ARA-derivative EETs. The analgesic effect of sEHI in LPS-treated rat was blocked by selective inhibitor CYP450. The sEHI and LPS-induced plasma EET/DHET ratio was not changed in the presence of selective inhibitor CYP450. Furthermore, the reduction of the plasma levels of prostaglandin E2 by sEHI did not influence the CYP450 in an LPS-treated rat [31]. Recently, studies pointed that CYP2J was distributed in cell bodies and 11, 12-EET reduced the neurotransmission in hippocampal CA1-CA3 area [32]. Thus, the sEHI and 14,15-EET increased the LTP and weather the effect the protein level or enzyme activity of CYP and sEH in hippocampus still needs further investigate.

LTP is a strengthened form of synaptic plasticity correlated with learning memory, aversion memory, and pain-related memory [33]. Inhibition of sEH causes increased levels of EETs [34, 35]. Recent experimentation in an animal model indicated pharmacological inhibition and deletion of a gene related to sEH in mice resilient to chronic social stress, suggesting that sEH may modulate synaptic function and regulate aversion memory [3]. Induction of LTP at the Schaffer collateral-CA1 synapses is NMDA receptor-dependent [10, 36, 37]. Increased tyrosine phosphorylation of both NR2A and NR2B is important for NMDA receptor activity and LTP formation [38–40]. Our results showed that selective NMDA receptor NR2B antagonist did not block HFS-induced LTP. Promotion of HFS-induced LTP at the SC-CA1 synapses by 14,15-EET and TPPU was blocked by an NR2B antagonist. Thus, NMDA receptor NR2B is involved in TPPU- and 14,15-EET-promoted hippocampal LTP. Immunoblot analysis showed that phosphorylation of NR2B at Tyr-1472 was increased by treatment with 14,15-EET and TPPU. Activation of Src family tyrosine kinase further enhanced the tyrosine phosphorylation of NR2B, which was sufficient for LTP induction [41, 42]. Increased Src family tyrosine kinase phosphorylation further enhanced NR2B phosphorylation and prevented NMDA receptor endocytosis to increase LTP induction [43]. Thus, TPPU and 14,15-EET increase NR2B (Tyr-1472) phosphorylation via Src family tyrosine kinase activation and enhance the NMDA receptor function of LTP. In addition, GluR1 phosphorylation at Ser831 is required for LTP induction [44, 45]. We also demonstrated that GluR1 phosphorylation was increased following sEHI and 14,15-EET treatments.

Evidence has implied involvement of adenylyl cyclase (AC), cAMP, and PKA in the late phase of LTP and spatial memory [15, 16, 46, 47]. The AC-mediated synaptic plasticity can be attributed to presynaptic promotion of neurotransmission release and postsynaptic cAMP response element binding protein (CREB) activation in the hippocampal region [48, 49]. cAMP is elevated by a PDE inhibitor, which may be engaged to block adenosine receptors to facilitate excitatory neurotransmitter release. We demonstrated that both TPPU- and 14,15-EET-induced potentiation effects on FSK-mediated LTP were prevented by the selective A1 adenosine receptor agonist, but were potentiated by the selective A1 adenosine receptor antagonist and PDE inhibitor at the hippocampal synapses. Thus, cAMP–PDE-adenosine signaling is involved in TPPU and 14,15-EET modulation of presynaptic glutamate release and plasticity at the hippocampal synapses [50, 51]. Interestingly, Inceoglu et al. also found that coinhibition of sEH and PDE4 activities by 1-trifluoromethoxyphenyl-3-(1-acetylpiperidin-4-yl) urea (TPAU) and rolipram, respectively, could stabilize epoxy fatty acids (EFAs) and increase epoxide/diol ratio, which lead to reduced pain-related responses [52]. Two possible mechanisms could explain why TPPU and 14,15-EET promoted FSK-mediated LTP in hippocampus. First, sEHI and 14,15-EET could increase cAMP level and promote LTP-mediated cAMP-PKA pathway. Second, the interaction between PDE inhibitor and sEHI caused higher levels of 14,15-EET resulting in enhanced induction and maintenance of LTP in the hippocampus [52]. Incubation with recombinant sEH, reduced EET level, has been demonstrated to reduce neutrophil migration in culture system [53]. In the future, we would like to investigate if the TPPU- and 14,15-EET-facilitated synaptic potentiation can be blocked by recombinant sEH in the hippocampus.

Local infusion with 14,15-EET into ventrolateral periaqueductal gray has antinociceptive effect in the Tail-flick test [54]. sEHI has been reported to be antihyperalgesia in inflammation pain and neuropathic pain [31, 55, 56]. Although modulation of cAMP signaling pathway is important for neuronal hyperexcitability and maintenance of hyperalgesia in pain [57, 58]. Inceoglu et al. have demonstrated that the importance of the steroid synthesis and cAMP are required for sEHI-mediated analgesia [31, 52]. Understanding the synaptic strength such as LTP of the spinal cord or cortex in pain pathway provides translational mechanisms in neuropathic pain [59]. We exhibited that sEHI and 14,15-EET facilitated HFS-LTP and FSK-LTP. We demonstrated that HFS-LTP- and FSK-LTP-increased EET-mediated synaptic neurotransmission by sEHI may provide the synaptic mechanism in pain pathway. Additionally, we showed that TPPU and 14,15-EET promoted FSK-mediated LTP and increased the phosphorylation of GluR1. Thus, cAMP/PKA activity is required for GluR1 phosphorylation-mediated GluR1 insertion into membranes for subsequent potentiation of the synaptic transmission in the presence of EETs [60, 61].

 Interaction between CaMKII and NR2B is required for synaptic plasticity. Activation of CaMKII leads to autophosphorylation at Thr-286 and induction of an increased association between CaMKII and NMDAR. The phosphorylation of NR2B at Tyr-1472 is critical for the binding of CaMKII to the NR2B subunit [62]. A sustained increase in CaMKII activity also induces phosphorylation of AMPA receptors or stargazine to promote binding of postsynaptic density (PSD)-95, which is necessary for maintaining LTP [11, 63–66]. We demonstrated that TPPU and 14,15-EET induced increased phosphorylation of CaMKII at Thr-286, phosphorylation of NR2B at Tyr-1472, and phosphorylation of GluR1 at Ser-831 to strengthen the glutamate-mediated synaptic plasticity. Moreover, evidence has shown that increased activity in the mitogen-activated protein kinase (MAPK) signaling cascade is associated with LTP in the hippocampal CA1 region and can be blocked by MEK inhibitor [67–71]. Ras-guanine nucleotide-releasing factor (Ras-GRF) is an activator of ERK MAPK, and the Ras/ERK MAPK cascade is required for LTP and memory processes [72–74]. The present study demonstrated that CaMKII is a key molecule in exogenous and endogenous EET-facilitated synaptic transmission and synaptic plasticity. Furthermore, activation of the ERK MAPK cascade may induce synaptic new protein, which is necessary for the maintenance of TPPU- and 14,15-EET-facilitated LTP in the hippocampus.

Overall, the study demonstrated that both sEHI and 14,15-EET combined with high-frequency stimulation trigger calcium influx through NMDA receptors, and the increased intracellular calcium resulted in activation of kinases, including CaMKII, PKA, and ERK. The enhanced phosphorylation of CaMKII, NR2B, and GluR1 produced increased hippocampal excitatory synaptic plasticity. The activated kinases, such as PKA and ERK, are translocated into the nucleus and initiate gene transcription and translation to maintain sEHI- and 14,15-EET-mediated synaptic potentiation (Figure 7).

## Abbreviations

2-CADO: 2-Chloroadenosine
14,15-EET: (±)14(15)-Epoxy-5Z,8Z,11Z-eicosatrienoic acid
20-HETE: 20-Hydroxy-5Z,8Z,11Z,14Z-eicosatetraenoic acid
ARA: Arachidonic acid
AMPA: *α*-amino-3-hydroxy-5-methyl-4-isoxazolepropionic acid
CaM: Ca2+/calmodulin
CaMKII: Ca2+/calmodulin-dependent protein kinase II
cLTP: Chemically induced LTP
cAMP: Cyclic AMP
CYP: Cytochrome P450
DHETs: Dihydroxyeicosatrienoic acids
DMSO: Dimethyl sulfoxide
DPCPX: Adenosine receptor antagonist 8-cyclopentyl-1,3-dipropylxanthine
EETs: Epoxyeicosatrienoic acids
EFA: Epoxy fatty acids
ERKs: Extracellular signal-regulated kinases
fEPSP: Field excitatory postsynaptic potential
FSK: Forskolin
HFS: High-frequency stimulation
KT5720: (9R,10S,12S)-2,3,9,10,11,12-Hexahydro-10-hydroxy-9-methyl-1-oxo-9,12-epoxy-1H-diindolo[1,2,3-fg:3′,2′,1′-kl]pyrrolo[3,4-i][1,6]benzodiazocine-10-carboxylic acid, hexyl ester
LTP: Long-term potentiation
LTD: Long-term depression
MAPK: Mitogen-activated protein kinase
NMDA: N-Methyl-D-aspartate
PKA: Protein kinase A
PSD: Postsynaptic density
Ras-GRF: Ras-guanine nucleotide-releasing factor
Ro 20-1724: 4-(3-Butoxy-4-methoxyphenyl)methyl-2-imidazolidone
Ro 25-6981: (*αR*,*βS*)-*α*-(4-hydroxyphenyl)-*β*-methyl-4-(phenylmethyl)-1-piperidinepropanol maleate
Rp-cAMPs: Rp-Cyclic 3′,5′-hydrogen phosphorothioate adenosine
sEH: Soluble epoxide hydrolase
TPPU: N-[1-(Oxopropyl)-4-piperidinyl]-N'-[4-(trifluoromethoxy) phenyl)-urea.
